# Impact of Spinal Instrumentation on Neurological Outcome in Patients with Intermediate Spinal Instability Neoplastic Score (SINS)

**DOI:** 10.3390/cancers14092193

**Published:** 2022-04-27

**Authors:** Moritz Lenschow, Maximilian Lenz, Niklas von Spreckelsen, Julian Ossmann, Johanna Meyer, Julia Keßling, Lukas Nadjiri, Sergej Telentschak, Kourosh Zarghooni, Peter Knöll, Moritz Perrech, Eren Celik, Max Scheyerer, Volker Neuschmelting

**Affiliations:** 1 Center for Neurosurgery, University of Cologne, 50937 Cologne, Germany; niklas.von-spreckelsen@uk-koeln.de (N.v.S.); jossmann@smail.uni-koeln.de (J.O.); sergej.telentschak@uk-koeln.de (S.T.); moritz.perrech@uk-koeln.de (M.P.); volker.neuschmelting@uk-koeln.de (V.N.); 2 Department of Orthopedics and Trauma Surgery, University of Cologne, 50937 Cologne, Germany; maximilian.lenz@uk-koeln.de (M.L.); johanna.meyer@uk-koeln.de (J.M.); julia.kessling@uk-koeln.de (J.K.); kourosh.zarghooni@uk-koeln.de (K.Z.); peter.knoell@uk-koeln.de (P.K.); max.scheyerer@uk-koeln.de (M.S.); 3 Department of Radiooncology and Cyberknife Center, University of Cologne, 50937 Cologne, Germany; lukas.nadjiri@uk-koeln.de (L.N.); eren.celik@uk-koeln.de (E.C.)

**Keywords:** spinal metastasis, spine tumor surgery, spinal instrumentation, instability, MESCC, SINS

## Abstract

**Simple Summary:**

Spinal epidural metastases are a common complication of malignancies that can compromise spinal stability and subsequently lead to neurologic deficits in addition to pain and overall reduced quality of life, often requiring spinal instrumentation. The spinal instability neoplastic score is an instrument used to evaluate spinal stability; a stable situation is assumed in cases of a SINS below 7 and instability in cases of a SINS above 12, but there is uncertainty in SINS 7 to 12. Our aim was to evaluate the benefit of spinal instrumentation in these cases in terms of neurological function in order to improve patient treatment.

**Abstract:**

Background: Adequate assessment of spinal instability using the spinal instability neoplastic score (SINS) frequently guides surgical therapy in spinal epidural osseous metastases and subsequently influences neurological outcome. However, how to surgically manage ‘impending instability’ at SINS 7–12 most appropriately remains uncertain. This study aimed to evaluate the necessity of spinal instrumentation in patients with SINS 7–12 with regards to neurological outcome. Methods: We screened 683 patients with spinal epidural metastases treated at our interdisciplinary spine center. The preoperative SINS was assessed to determine spinal instability and neurological status was defined using the Frankel score. Patients were dichotomized according to being treated by instrumentation surgery and neurological outcomes were compared. Additionally, a subgroup analysis of groups with SINS of 7–9 and 10–12 was performed. Results: Of 331 patients with a SINS of 7–12, 76.1% underwent spinal instrumentation. Neurological outcome did not differ significantly between instrumented and non-instrumented patients (*p* = 0.612). Spinal instrumentation was performed more frequently in SINS 10–12 than in SINS 7–9 (*p* < 0.001). The subgroup analysis showed no significant differences in neurological outcome between instrumented and non-instrumented patients in either SINS 7–9 (*p* = 0.278) or SINS 10–12 (*p* = 0.577). Complications occurred more frequently in instrumented than in non-instrumented patients (*p* = 0.016). Conclusions: Our data suggest that a SINS of 7–12 alone might not warrant the increased surgical risks of additional spinal instrumentation.

## 1. Introduction

An important principle in the management of patients presenting with spinal epidural osseous metastases is the assessment of spinal stability to evaluate the benefit of spinal instrumentation prior to radiation and/or systemic treatment. The spinal instability neoplastic score (SINS) has been developed as a prognostic decision-making tool to identify patients who may benefit from spinal stabilizing intervention and, thus, potentially prevent neurological deterioration [1].

The SINS, which has found wide application among spine surgeons and oncological clinicians, sums up six clinical and radiographic parameters individually scored with 1 to 3 points: (1) location, (2) mechanical pain, (3) bone lesion quality, (4) spinal alignment, (5) vertebral body collapse and (6) posterior involvement of spinal elements [2]. A stable spine was found to be assumed in the case of a SINS value of less than 7 and an unstable spine in the case of a value of more than 12. However, uncertainty regarding the need for spinal instrumentation exists in the case of a SINS value of 7 to 12, thus defined as intermediate instability. How to manage intermediate instability is controversially discussed among clinicians and the benefit of surgical intervention outweighing the potential risk of surgical complications in those patients is as yet unknown [3,4].

Due to this ambiguity, a reclassification into a stable (SINS 0 to 9) and an unstable (SINS 10 to 18) group has been suggested [5,6,7].

The purpose of this study was to retrospectively evaluate the clinical utility of spinal instrumentation in SINS 7 to 12 as well as the utility of a reclassification based on SINS ≥ 10 in terms of neurological outcome.

## 2. Materials and Methods

### 2.1. Data Collection

For this retrospective analysis, we reviewed all consecutive patients admitted to our interdisciplinary spine center between March 2009 and March 2021 who were treated for spinal osseous metastases. All data were obtained from the centers’ electronic medical record and imaging database. Study approval was obtained by the local ethics committee (approval code: 20-1643).

All patients who initially presented with a SINS of 7 to 12 were included in this study. Patients were excluded in the case of missing or incomplete data records and in the case of omitted treatment (e.g., palliative and best supportive care). In the case of multiple spine lesions, the SINS was determined for each treated lesion and the respective maximum SINS was reported for the case. Patients with multiple spinal metastatic lesions that underwent both instrumentation and non-instrumentation techniques were excluded from the study.

The following parameters were recorded: age, gender, Karnofsky Performance Status (KPS), primary tumor origin, epidural spinal cord compression (ESCC) scale and medical comorbidities (diabetes mellitus, coronary heart disease, history of smoking and chronic obstructive pulmonary disease (COPD), history of deep vein thrombosis, obesity (defined as a body mass index > 30), Osteoporosis and current glucocorticoid therapy at the time of treatment) [8,9]. Multiple myeloma and lymphoma were summarized as hematopoietic cancers.

Regarding treatment-related complications, we recorded the following: wound healing disorders, wound infections, spondylodesis dislocation or failure, thrombosis and pneumonia.

Spinal instability as well as individual treatment strategies were determined by the treating surgeon or tumor board panel. All patients were primarily treated by (a) decompressive surgery, (b) decompressive surgery and instrumentation, (c) instrumentation without decompression, (d) vertebroplasty or (e) local radiotherapy. In general, spinal decompression surgery was performed in all cases of (impending) epidural cord compression, except for highly radiosensitive tumors, and in any case of neurologic impairment. There were no institutional protocols for spinal instrumentation; decisions were made on a case-by-case basis based on patient- and case-specific findings and the overall assessment of stability by the treating senior spine surgeon.

The prerequisite for all surgical procedures in this study was a performance status sufficient for postoperative recovery and the availability of systemic treatment options for the postoperative period. Surgical interventions were omitted in cases with a severely limited prognostic assessment by the treating oncologist, e.g., an expected survival of less than one month.

All patients were scheduled for adjuvant radiotherapy and systemic oncological therapy.

Depending on the treatment strategy, patients were dichotomized into an instrumentation and non-instrumentation group. Further subgroup analysis was done to compare instrumented and non-instrumented patients with SINS values of 7 to 9 and 10 to 12.

### 2.2. SINS and ESCC Assessment

The SINS and ESCC scores were radiographically assessed based on preoperative computed tomography images and magnetic resonance imaging of the corresponding spine as previously described [1,9]. The minimum requirements for all computed tomography scans included axial and sagittal slices of at least 5 mm thickness as well as bone and soft tissue windows. Magnetic resonance imaging included T1-weighted sequence before and after intravenous injection of a gadolinium-based contrast-enhancing agent as well as at least 5 mm thick T2-weighted sequences.

Medical charts and filed medical history were reviewed to assess the pain component of the SINS, mechanical pain was defined as pain due to movement or loading that did not improve with recumbency.

### 2.3. Assessment of Neurological Function

Neurological status was assessed at the initial examination and at last follow-up and classified according to the Frankel score [10]. A patient was deemed ambulatory in the case of a Frankel score of D and E. The neurological outcome was assessed based on changes in pre- and postoperative Frankel scores and defined as follows: improvement (increase of at least one grade), stable (no change) or worsening (decrease of at least one grade).

### 2.4. Statistical Analysis

Clinical characteristics are displayed using descriptive statistics. Categorical variables were compared by a Chi-Square and a Fisher’s Exact test, when appropriate. Continuous variables were tested for normal distribution using the Kolmogorov–Smirnov test and for homoscedasticity using the White test. Data are reported as mean ± standard deviation or median (95% confidence interval). Group means from normally distributed data were compared using a two-sided unpaired Student’s t-test while a Mann–Whitney U test was used in the case of non-normal or heteroscedastic distribution of data. All calculations were performed using SPSS software (Version 27, IBM SPSS Statistics for Windows, Armonk, NY, USA). A *p*-value < 0.05 was considered statistically significant.

## 3. Results

During the study period, a total of 683 patients with spinal epidural metastases were treated at our spine center. Of those, 331 (48.5%) presented with a SINS value of 7 to 12 and were included in the study. An additional 69 patients with a SINS value of 7 to 12 were excluded due to incomplete data records (*n* = 63), omitted treatment (*n* = 5) or treatment including both instrumentation and non-instrumentation techniques (*n* = 1).

Median follow-up was 3 (2 to 4) months.

### 3.1. Patient Characteristics

**Figure 1:**
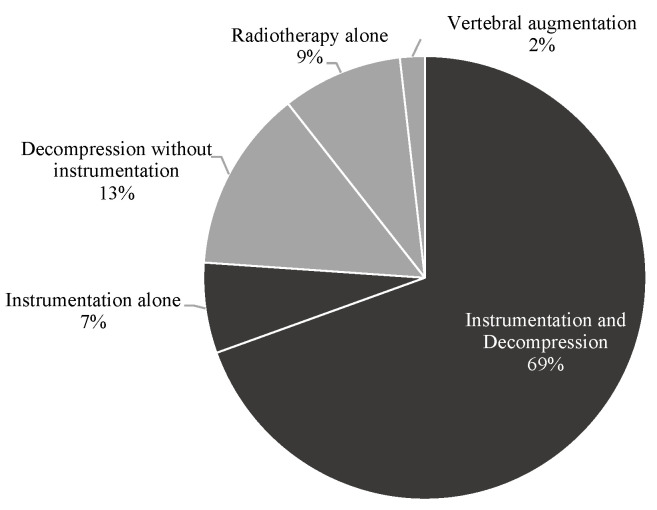
Treatment modalities in the study cohort.

According to the respective therapy strategy, 252 (76.1%) patients underwent spinal instrumentation surgery and 79 (23.9%) were assigned to the non-instrumentation group (see Figure 1). Within the instrumentation group, 230 (91.3%) patients were treated with spinal instrumentation and decompression and 22 (8.7%) with spinal instrumentation only without decompression. Within the non-instrumentation group, stand-alone decompressive surgery was performed in 44 (55.7%) patients, vertebral augmentation in 6 (7.6%) and primary radiotherapy in 29 (36.7%) patients. Overall, surgical decompression was performed in 274 (82.8%) patients prior to radiation treatment.

Median patient age was 64 (62 to 66) years, 35.0% were female. Median KPS was 60 (60 to 70). Most metastases originated from the lung (18.7%), prostate (17.2%) and breast (15.1%).

Female gender (*p* = 0.048) and a history of thrombosis (*p* = 0.030) were significantly more frequent in the non-instrumented group than among the instrumented patients, the groups did not differ with respect to age (*p* = 0. 558), KPS (*p* = 0.325), tumor origin (*p* = 0.399), and other medical comorbidities (all *p* > 0.1). Detailed information regarding patient characteristics including medical conditions is provided in Table 1.

### 3.2. Spinal Instability and Epidural Spinal Cord Compression

The SINS was assessed as a sum score of 7 to 9 in 140 (42.3%) and 10 to 12 in 191 (57.7%) of all patients. A SINS value of 10 to 12 was significantly more frequent in instrumented (65.1%) than non-instrumented patients (34.2%; *p* < 0.001).

Regarding the SINS score components (see Table 2), the bone lesion quality was found to significantly differ between the two groups (*p* = 0.001) with more frequent osteolytic lesions found in the instrumented (84.1%) than in the non-instrumented (65.8%) patients. The presence and degree of vertebral body collapse was higher in the instrumented group (>50% vertebral body collapse in 34.5%) than in the non-instrumented group (>50% vertebral body collapse in 12.7%; *p* < 0.001). The two groups did not differ with regards to the SINS components ‘mechanical pain’ (*p* = 0. 052), ‘location’ (*p* = 0.565), ‘vertebral alignment’ (*p* = 0.108) or ‘involvement of the posterior elements’ (*p* = 0.743).

The distribution of ESCC values in the instrumented and non-instrumented group is displayed in Table A1 in Appendix A. High grade epidural spinal cord compression (ESCC 2 and 3) was present in 63.4% of all cases and showed a significantly higher prevalence in the instrumented (69.0%) than in the non-instrumented group (45.6%; *p* = 0.001).

### 3.3. Neurological Outcome

**Figure 2:**
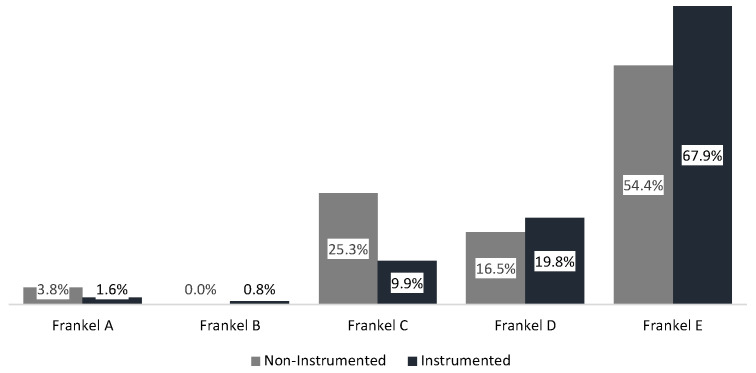
Frankel score at initial examination.

At first examination, the majority of patients presented with a Frankel score of E (64.7%). Frankel scores at first examination were significantly higher in the instrumented than in the non-instrumented group (*p* = 0.007; see Figure 2 and Table A2 in Appendix A). Walking ability (Frankel score D and E) was preserved in 83.7% of all patients at first examination, with a higher proportion of initially ambulatory patients in the instrumented (87.7%) than in the non-instrumented group (70.9%; *p* = 0.001).

At last follow-up, the Frankel score had improved in 17.8% (*n* = 59), remained stable in 75.8% (*n* = 251) and worsened in 6.3% (*n* = 21) of all patients. Of the 54 patients initially unable to walk, 28 (51.9%) regained ambulation following treatment, whereas 12/21 (57.1%) patients who experienced neurological worsening lost their ability to walk. Neurological worsening was due to local tumor recurrence (*n* = 5) or distant tumor progression (*n* = 12) not amendable to further treatment due to reduced performance status or cerebral progression (*n* = 4).

**Figure 3:**
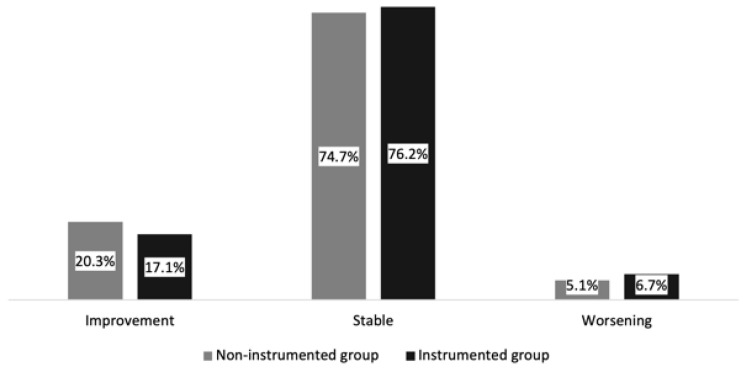
Changes in Frankel score: the instrumented and non-instrumented groups in the study cohort.

Changes in Frankel score (*p* = 0.730) or ambulation status (*p* = 0.555) were not significantly different between the instrumented and non-instrumented group (see Table 3 and Figure 3).

### 3.4. Patient Cross over from the Non-Instrumented to the Instrumented Group

One patient underwent spinal instrumentation 28 days following initial decompressive surgery for progressive mechanical pain and a new local kyphotic deformity.

### 3.5. Complications

Complications are listed in Table 4. Overall, complications occurred in 43 (13.0%) cases and were more frequent in instrumented (15.5%) than in non-instrumented patients (5.1%; *p* = 0.016). The most frequent complications were wound healing disorders (9.1%) and spondylodesis dislocation/failure (5.4%). Of note, thrombosis (2.4%) or pneumonia (3.6%) occurred only in the group of instrumented patients in our study cohort.

### 3.6. Subgroup Analysis of Neurological Outcome in Patients with SINS Score 7 to 9 and 10 to 12

There were no significant differences between the groups of SINS 7 to 9 and SINS 10 to 12 in terms of patient characteristics (all *p* > 0.05), ESCC score (*p* = 0.105) or neurological function at first examination (*p* = 0.135; see Table A3 in Appendix A). Regarding the applied treatment strategy, the subgroups significantly differed: the group of patients with a SINS of 7 to 9 were less frequently instrumented (62.9%) than the corresponding group of patients with a SINS of 10 to 12 (85.9%; *p* < 0.001, see Table 5).

Within the subgroup of SINS 7 to 9 (see Table A4 in Appendix A), neurological function at first examination was less severely affected in the instrumentation (86.4% preserved walking ability) than in the non-instrumentation group (65.4% preserved walking ability; *p* = 0.003).

Neurologic outcome in this subgroup, in terms of changes in Frankel score and ambulatory status, did not differ significantly between the instrumentation and non-instrumentation group (*p* = 0.577 and *p* = 0.727, respectively).

The subgroup of SINS 10 to 12 (see Table A5 in Appendix A) showed no significant difference between the instrumentation and non-instrumentation group in terms of neurological function at first examination (*p* = 0.941), changes in Frankel score (*p* = 0.278) or ambulatory status (*p* = 0.535).

### 3.7. Subgroup Analysis of Neurological Outcome in Instrumented or Non-Instrumented Patients Presenting with SINS Scores 7 to 9 or 10 to 12

For neurologic outcome in all instrumented patients, there was no significant difference between patients with a SINS of 7 to 9 and patients with a SINS of 10 to 12 in terms of changes in Frankel score (*p* = 0.418) or ambulatory status (*p* = 0.811; see Table A6 in Appendix A).

Comparison of the SINS 7 to 9 with the SINS 10 to 12 group in all non-instrumented patients showed no significant differences in changes in Frankel score (*p* = 0.332) or ambulatory function (*p* = 0.440; see Table A7 in Appendix A).

## 4. Discussion

The criteria for the assessment of spinal integrity and the clinical need for spinal instrumentation in the case of intermediate instability (SINS 7 to 12), the most prevalent SINS category in clinical practice, are unclear, and both spinal instrumentation as well as non-stabilizing treatment methods are regularly employed [3,7,11,12,13]. The aim of this study was to assess the utility of spinal instrumentation in the case of intermediate instability and the utility of a SINS reclassification in terms of neurological function.

In the case of spinal metastasis, neurological function has a critical impact on quality of life and overall survival [4,14]. Neurological function depends on spinal stability, which in turn is defined by pain, deformity, and neurological function under physiological load [15,16]. While the effects of spinal instrumentation on pain reduction in the SINS 7 to 12 subgroup have been shown previously [17], this is the first study to examine the effects of spinal instrumentation on neurological function, as well as the largest series to date with a specific focus on impending instability.

Since spinal instrumentation serves both as an adjunctive measure to restore impaired neurological function and as a protective measure to prevent secondary instability-related neurological impairment, both neurologically intact and impaired patients were included in this study.

Overall, neurological function improved or stabilized in 93.1% of all patients and walking ability was restored in 51.9% of all initially non-ambulatory patients. As this is most likely due to the surgical decompression (82.8% of all cases) or radiotherapy in cases of radiosensitive tumors, we found no significant difference between the instrumented and non-instrumented groups with regard to post-therapeutic changes in either Frankel score or ambulatory status [18].

While this finding is in support of the recommendations by Ivanishvili et al. not to perform spinal instrumentation in the case of a SINS below 12, it is in contrast with a study by Hussain et al., who reported significantly improved patient-reported outcomes including functional aspects in the case of instrumentation in SINS 7 to 12 [6,19]. However, since disability severity was significantly associated with the presence of mechanical pain in their study, these results may primarily reflect an improvement in instability-related pain with spinal instrumentation, but may not translate to the neurological function we studied.

Furthermore, treatment-associated complications need to be taken into account, as our study confirms an increased complication-risk in the case of spinal instrumentation, potentially affecting overall survival [4,20,21,22,23].

Due to the therapeutic uncertainty in the case of a SINS of 7 to 12 and to allow for more uniform treatment strategies in this large cohort of patients, previous authors suggested elimination of the intermediate category and dichotomization of the SINS into a stable (SINS 0 to 9) and an unstable (SINS 10 to 18) category, based on retrospective analyses of respective treatment patterns and patient-reported outcome measures [5,6,7].

Applying this reclassification in our cohort, while confirming more frequent instrumentation in SINS 10 to 12 in clinical practice [5], we observed no benefit from additional instrumentation in terms of neurological outcome in the respective subgroups of either SINS 7 to 9 or SINS 10 to 12.

Thus, although differences between SINS 7 to 9 and 10 to 12 in terms of clinical outcome following spinal instrumentation have been reported, in terms of neurological function assessed according to the Frankel grade, our findings do not support the concept of SINS 7 to 9 and 10 to 12 to differentiate spinal (in)stability, as neither subgroup in our cohort neurologically benefited from additional instrumentation [6,17].

In this regard, although we did not find an advantage of instrumentation in terms of neurological outcome in either SINS 7 to 12 or the respective subgroups, our study may be limited by its focus on the overall SINS at initial presentation, as spinal instrumentation may be deemed necessary or precluded regardless of the SINS value depending on several factors. In our study, lytic bone lesion and a higher extent of vertebral body collapse were significantly associated with spinal instrumentation, possibly indicating greater clinical emphasis on qualitative components of the SINS (e.g., parameters of lesion morphology) over less significant risk factors (e.g., location) [3,5]. Furthermore, iatrogenic instability due to extensive decompressive surgery in the case of higher-grade epidural compression might warrant instrumentation regardless of SINS grading to preserve or restore neurological function, which is in line with higher ESCC grades in the instrumentation group in our study [5,6], and spinal instrumentation may be performed irrespective of other factors to alleviate mechanical pain, although we observed no significant association with the respective SINS component in our cohort [24,25,26]. Therefore, to avoid inappropriate surgical treatment approaches, surgery in our study was not considered on the basis of prognostic assessments, but rather depending on the availability and clinical applicability of adjuvant systemic therapy to provide continuous postoperative systemic tumor control [27,28,29,30].

Our study carries several limitations. Firstly, due to its retrospective, single-center design as well as the heterogeneity of patients and treatment strategies. Secondly, the 5-mm slice thickness set as the minimum imaging requirement was based on the imaging and staging approach common in many radiology practices outside our institution, possibly resulting in an underestimation of the overall metastatic disease burden; however, a significant impact of these small metastases on the values examined in the study seems unlikely. Thirdly, the effects of pharmaceutical approaches, such as osteoclast inhibitors, were not analyzed as we considered the evaluation of conservative (preventive) measures to be outside the scope of this study. Prospective randomized trials are warranted to further investigate the utility of spinal instrumentation in cases of impending instability.

## 5. Conclusions

Although spinal instrumentation was the main surgical procedure in our cohort of patients initially presenting with a SINS of intermediate instability (7 to 12) in our retrospective data analysis, we found no benefit in neurologic outcome compared to corresponding SINS-scored patients that underwent decompressive surgery alone or primary radiation, even in the subgroup of SINS 10 to 12.

The SINS score seems to be limited in that regard to identify the patients with intermediately scored SINS that may still benefit from additional instrumentation. Accordingly, with the increased complication risk that the additionally instrumented patients carry, our data suggest that treatment without instrumentation should be reconsidered, opting for further prospective randomized trials in patients with intermediate scored SINS.

## Figures and Tables

**Table 1:** Patient characteristics.

Patient Characteristics [*n*, %]	Non-Instrumented (*n* = 79)	Instrumented (*n* = 252)	*p*-Value
Age in years [median; 95% CI]	64 (61–69)	63 (62–66)	0.558
Female gender	35 (44.3%)	81 (32.1%)	0.048
KPS [median; 95% CI]	60 (50–70)	60 (60–70)	0.109
Histology			0.345
Lung	14 (17.7%)	48 (19.0%)	
Prostate	17 (21.5%)	40 (15.9%)	
Breast	16 (20.3%)	34 (13.5%)	
Hematologic	9 (11.4%)	32 (12.7%)	
Renal	7 (8.9%)	22 (8.7%)	
Gastrointestinal	5 (5.3%)	23 (9.1%)	
Thyroid	0 (0.0%)	12 (4.8%)	
Unknown primary	4 (5.1%)	6 (2.4%)	
Mesenchymal tissue	2 (2.5%)	5 (2.0%)	
Bladder	0 (0%)	6 (2.4%)	
Skin	0 (0%)	4 (1.6%)	
Others	5 (6.3%)	20 (7.9%)	
Comorbidities			
Smoking/COPD	19 (24.1%)	64 (25.4%)	0.882
CHD/arteriosclerosis	8 (10.1%)	42 (16.7%)	0.207
Diabetes mellitus	12 (15.2%)	36 (14.3%)	0.855
Obesity	13 (16.5%)	26 (10.3%)	0.140
Thrombosis	9 (11.4%)	11 (4.4%)	0.030
Glucocorticoid medication	7 (8.9%)	12 (4.8%)	0.174
Osteoporosis	4 (5.1%)	6 (2.4%)	0.258

**Table 2:** Spinal instability neoplastic score components.

SINS Components [*n*, %]	Non-Instrumented (*n* = 79)	Instrumented (*n* = 252)	*p*-Value
Spine location			0.461
Junctional	49 (62.0%)	139 (55.2%)	
Mobile spine	9 (11.4%)	47 (18.7%)	
Semirigid	21 (26.6%)	66 (26.2%)	
Rigid	0.0 (0.0%)	0.0 (0.0%)	
Pain			0.052
Mechanical pain	43 (54.4%)	168 (66.7%)	
Occasional pain	22 (27.8%)	52 (20.6%)	
Painless lesion	14 (17.7%)	32 (12.7%)	
Bone lesion quality			0.001
Lytic	52 (65.8%)	212 (84.1%)	
Mixed lytic/blastic	19 (24.1%)	25 (9.9%)	
Blastic	8 (10.1%)	15 (6.0%)	
Spinal alignment			0.075
Subluxation/translation	0.0 (0.0%)	0.0 (0.0%)	
De novo deformity	6 (7.6%)	39 (15.5%)	
Normal alignment	73 (92.4%)	213 (84.5%)	
Vertebral body collapse			< 0.001
>50% collapse	10 (12.7%)	87 (34.5%)	
<50% collapse	19 (24.1%)	26 (10.3%)	
No collapse with >50% body involved	28 (35.4%)	116 (46.0%)	
None of the above	22 (27.8%)	23 (9.1%)	
Posterior involvement of spinal elements			0.743
Bilateral	40 (50.6%)	110 (43.7%)	
Unilateral	26 (32.9%)	119 (47.2%)	
None of the above	13 (16.5%)	23 (9.1%)	

**Table 3:** Changes in Frankel score and ambulatory status between initial examination and last follow-up.

Neurological Status [*n*, %]	Non-Instrumented (*n* = 79)	Instrumented (*n* = 252)	*p*-Value
Frankel score			0.730
Improvement	16 (20.3%)	43 (17.1%)	
Stable	59 (74.7%)	192 (76.2%)	
Worsening	4 (5.1%)	17 (6.7%)	
Ambulatory status			0.555
Regained ability to walk	9 (11.4%)	19 (7.5%)	
Unchanged	67 (84.8%)	224 (88.9%)	
Lost ability to walk	3 (3.8%)	9 (3.6%)	

**Table 4:** Complications.

Complications [*n*, %]	Non-Instrumented (*n* = 79)	Instrumented (*n* = 252)	*p*-Value
Wound healing disorder	4 (5.1%)	26 (10.3%)	0.156
Wound infection	0 (0.0%)	10 (4.0%)	0.125
Material dislocation/failure	1 (1.3%)	17 (6.7%)	0.085
Thrombosis	0 (0.0%)	6 (2.4%)	0.342
Pneumonia	0 (0.0%)	9 (3.6%)	0.121

**Table 5:** Treatment strategies within the subgroups SINS 7–9 and SINS 10–12.

Treatment Strategies [*n*, %]	SINS 7–9 (*n* = 140)	SINS 10–12 (*n* = 191)	*p*-Value
Instrumentation			<0.001
Instrumentation and decompression	78 (55.7%)	152 (79.6%)	
Instrumentation without decompression	10 (7.1%)	12 (6.3%)	
Non-instrumentation			
Decompression	34 (24.3%)	10 (5.2%)	
Vertebroplasty	2 (1.4%)	4 (2.1%)	
Radiotherapy	16 (11.4%)	13 (6.8%)	

## Data Availability

The datasets generated and/or analyzed in this study are available upon reasonable request.

## Appendix A

**Table A1:** Epidural spinal cord compression score.

ESCC Score [*n*, %]	Non-Instrumented (*n* = 79)	Instrumented (*n* = 252)	*p*-Value
0	17 (21.5%)	9 (3.6%)	0.001
1a	3 (3.8%)	9 (3.6%)	
1b	15 (19.0%)	20 (7.9%)	
1c	8 (10.1%)	40 (15.9%)	
2	10 (12.7%)	84 (33.3%)	
3	26 (32.9%)	90 (35.7%)	

**Table A2:** Frankel score at initial examination.

Frankel Score [*n*, %]	Non-Instrumented (*n* = 79)	Instrumented (*n* = 252)	*p*-Value
A	3 (3.8%)	4 (1.6%)	0.005
B	0 (0.0%)	3 (1.2%)	
C	20 (25.3%)	25 (9.9%)	
D	13 (16.5%)	49 (19.4%)	
E	43 (54.4%)	171 (67.9%)	

**Table A3:** Comparison of the subgroups SINS 7 to 9 and SINS 10 to 12.

Parameter [*n*, %]	SINS 7–9 (*n* = 140)	SINS 10–12 (*n* = 191)	*p*-Value
Age in years [median; 95% CI]	64 (59–67)	63 (62–67)	0.293
Female Gender	42 (30.0%)	74 (38.7%)	0.104
KPS [median; 95% CI]	60.0 (60–70)	60.0 (60–70)	0.504
Histology			0.091
Lung	30 (21.4%)	32 (16.8%)	
Prostate	28 (20.0%)	29 (15.2%)	
Breast	19 (13.6%)	31 (16.2%)	
Hematologic	15 (10.7%)	26 (13.6%)	
Renal	10 (7.1%)	19 (9.9%)	
Gastrointestinal	7 (5.0)	21 (11.0%)	
Thyroid	4 (2.9%)	8 (4.2%)	
Unknown primary	5 (3.6%)	5 (2.6%)	
Mesenchymal tissue	7 (5.0%)	0 (0.0%)	
Bladder	2 (1.4%)	4 (2.1%)	
Skin	2 (1.4%)	2 (1.0%)	
Others	11 (7.9%)	14 (7.3%)	
Comorbidities			
Smoking/COPD	34 (24.3%)	49 (25.7%)	0.777
CHD/arteriosclerosis	23 (16.4%)	27 (14.1%)	0.565
Diabetes mellitus	17 (12.2%)	31 (16.2%)	0.297
Obesity	18 (12.9%)	21 (11.0%)	0.604
Thrombosis	7 (5.0%)	13 (6.8%)	0.496
Glucocorticoid medication	8 (5.7%)	11 (5.8%	0.986
Osteoporosis	2 (1.4%)	8 (4.2%)	0.147
ESCC score			0.004
0	19 (13.6%)	7 (3.7%)	
1a	7 (5.0%)	5 (2.6%)	
1b	17 (12.1%)	18 (9.4%)	
1c	13 (9.3%)	35 (18.3%)	
2	36 (25.7%)	58 (30.4%)	
3	48 (34.3%)	68 (35.6%)	
Initial Frankel score			0.135
A	5 (3.6%)	2 (1.0%)	
B	1 (0.7%)	2 (1.0%)	
C	24 (17.1%)	21 (11.0%)	
D	29 (20.7%)	33 (17.3%)	
E	81 (7.9%)	133 (69.6%)	

**Table A4:** Frankel score and ambulatory status in instrumented and non-instrumented patients with SINS 7 to 9.

Neurological Status [*n*, %]	Non-Instrumented (*n* = 52)	Instrumented (*n* = 88)	*p*-Value
Initial Frankel score			0.035
A	3 (5.8%)	2 (2.3%)	
B	0 (0.0%)	1 (1.1%)	
C	15 (28.8%)	9 (10.2%)	
D	10 (19.2%)	19 (21.6%)	
E	24 (46.2%)	57 (64.8%)	
Changes in Frankel score			0.577
Improvement	10 (19.2%)	12 (13.6%)	
Stable	38 (73.1%)	71 (80.7%)	
Worsening	4 (7.7%)	5 (5.7%)	
Initially preserved ambulation	34 (65.4%)	76 (86.4%)	0.003
Changes in ambulatory status			0.727
Regained ability to walk	6 (11.5%)	7 (8.0%)	
Unchanged	43 (82.7%)	77 (87.5%)	
Lost ability to walk	3 (5.8%)	4 (4.5%)	

**Table A5:** Frankel score and ambulatory status in instrumented and non-instrumented patients with SINS 10 to 12.

Neurological Status [*n*, %]	Non-Instrumented (*n* = 27)	Instrumented (*n* = 164)	*p*-Value
Initial Frankel score			0.941
A	0 (0.0%)	2 (1.2%)	
B	0 (0.0%)	2 (1.2%)	
C	5 (18.5%)	16 (9.8%)	
D	3 (11.1%)	30 (18.3%)	
E	19 (70.4%)	114 (69.5%)	
Changes in Frankel score			0.278
Improvement	6 (22.2%)	30 (18.3%)	
Stable	21 (77.8%)	120 (73.2%)	
Worsening	0 (0.0%)	14 (8.5%)	
Initially preserved ambulation	22 (81.5%)	144 (87.8%)	0.361
Changes in ambulatory status			0.535
Regained ability to walk	3 (11.1%)	12 (7.4%)	
Unchanged	24 (88.9%)	147 (89.6%)	
Lost ability to walk	0 (0.0%)	5 (3.0%)	

**Table A6:** Frankel score and ambulatory status in instrumented patients with SINS 7 to 9 and SINS 10 to 12.

Neurological Status [*n*, %]	SINS 7–9 (*n* = 88)	SINS 10–12 (*n* = 164)	*p*-Value
Initial Frankel score			0.921
A	2 (2.3%)	2 (1.2%)	
B	1 (1.1%)	2 (1.2%)	
C	9 (10.2%)	16 (9.8%)	
D	19 (21.6%)	30 (18.3%)	
E	57 (64.8%)	114 (69.5%)	
Changes in Frankel score			0.418
Improvement	13 (14.8%)	30 (18.3%)	
Stable	71 (80.7%)	121 (73.8%)	
Worsening	4 (4.5%)	13 (7.9%)	
Initially preserved ambulation	77 (87.5%)	144 (87.8%)	0.944
Changes in ambulatory status			0.811
Regained ability to walk	7 (8.0%)	12 (7.3%)	
Unchanged	77 (87.5%)	147 (89.6%)	
Lost ability to walk	4 (4.5%)	5 (3.0%)	

**Table A7:** Frankel score and ambulatory status in all non-instrumented patients with SINS 7 to 9 and SINS 10 to 12.

Neurological Status [*n*, %]	SINS 7–9 (*n* = 52)	SINS 10–12 (*n* = 27)	*p*-Value
Initial Frankel score			0.177
A	3 (5.8%)	0 (0.0%)	
B	0 (0.0%)	0 (0.0%)	
C	15 (28.8%)	5 (18.5%)	
D	10 (19.2%)	3 (11.1%)	
E	24 (87.5%)	19 (70.4%)	
Changes in Frankel score			0.332
Improvement	10 (19.2%)	6 (22.2%)	
Stable	38 (73.1%)	21 (77.8%)	
Worsening	4 (7.7%)	0 (0.0%)	
Initially preserved ambulation	34 (65.4%)	22 (81.5%)	0.135
Changes in ambulatory status			0.440
Regained ability to walk	6 (11.5%)	3 (11.1%)	
Unchanged	43 (82.7%)	24 (88.9%)	
Lost ability to walk	3 (5.8%)	0 (0.0%)	
